# PD-L1 Expression Is an Independent Marker for Lymph Node Metastasis in Middle Eastern Endometrial Cancer

**DOI:** 10.3390/diagnostics11030394

**Published:** 2021-02-25

**Authors:** Abdul K. Siraj, Sandeep Kumar Parvathareddy, Padmanaban Annaiyappanaidu, Nabil Siraj, Maha Al-Rasheed, Ismail A. Al-Badawi, Fouad Al-Dayel, Khawla S. Al-Kuraya

**Affiliations:** 1 Human Cancer Genomic Research, Research Center, King Faisal Specialist Hospital and Research Center, P.O. Box 3354, Riyadh 11211, Saudi Arabia; asiraj@kfshrc.edu.sa (A.K.S.); psandeepkumar@kfshrc.edu.sa (S.K.P.); pannaiyappanaidu97@kfshrc.edu.sa (P.A.); nabilsiraj1991@hotmail.com (N.S.); mrasheed@kfshrc.edu.sa (M.A.-R.); 2 Department of Obstetrics-Gynecology, King Faisal Specialist Hospital and Research Center, P.O. Box 3354, Riyadh 11211, Saudi Arabia; ibadawi@kfshrc.edu.sa; 3 Department of Pathology, King Faisal Specialist Hospital and Research Centre, P.O. Box 3354, Riyadh 11211, Saudi Arabia; dayelf@kfshrc.edu.sa

**Keywords:** PD-L1, endometrial cancer, lymph node metastasis, immunohistochemistry

## Abstract

Programmed death ligand 1 (PD-L1) expression in endometrial cancer (EC) tumor cells have been reported in several studies with inconsistent results. Furthermore, there is scarcity of data on the prevalence and prognostic significance of PD-L1 expression in EC from Middle Eastern ethnicity. We aimed to assess PD-L1 expression in a large cohort of Middle Eastern EC and to correlate this with clinico-pathological factors, as well as mismatch repair (MMR) protein status and patients’ outcome. PD-L1 expression was investigated using immunohistochemistry on tissue microarray in an unselected cohort of 440 EC. Kaplan–Meier and logistic regression analysis were used to compare the outcome and prognostic factors. PD-L1 expression in tumor tissue was detected in 18.9% (83/440) EC cases with no impact on survival. When stratified for MMR protein status, PD-L1 expression was similar for both MMR deficient and MMR proficient ECs. However, the expression of PD-L1 in tumor cells was significantly associated with type II (non-endometrioid) histology (*p* = 0.0005) and lymph node metastasis (*p* = 0.0172). Multivariate analysis showed PD-L1 expression to be an independent risk factor for lymph node metastasis (odds ratio: 2.94; 95% CI: 1.26–6.84; *p* = 0.0123). In conclusion, PD-L1 was strongly associated with non-endometrioid EC and was an independent prognostic marker of lymph node metastasis.

## 1. Introduction

Endometrial cancer (EC) is the most common gynecological malignancy in the Western countries [1,2]. Similarly, in Saudi Arabia, EC is the most common gynecological cancer and the fourth most common cancer among Saudi women [3]. The incidence is increasing in Saudi Arabia, which could be partially attributed to the high prevalence of obesity in this population [3,4,5]. Although most cases present at early stage and have a good prognosis, about 15–20% of patient present with advanced stage and aggressive morphology [6,7]. Recurrence and metastasis remain a challenge despite the available therapeutic options of surgery, chemotherapy, radiotherapy, and hormonal therapy.

Recently, immune checkpoint inhibitor has emerged as a promising therapeutic option in oncology and has significantly altered the standard treatment for many advanced cancers [8]. The most common mechanisms underlying immunotherapy are programmed cell death protein-1 (PD-1) and programmed death ligand-1 (PD-L1) which serve as immune checkpoints in tumor micro environment [9,10]. The PD-1/PD-L1 axis is a critical immune modulatory pathway that inhibits the immune reaction to cancer cells by negatively regulating T-cell functions [10,11].

After the Food and Drug Administration (FDA) approval of PD-L1 inhibitors for treatment of microsatellite instable and metastatic cancers in several types of malignancies including EC [12,13,14,15,16], this promising therapeutic modality has been the encouraging force behind the exploration of PD-L1 expression in EC in several recent reports [17,18,19,20,21,22]. However, results from these studies were not consistent. The discrepancies between these reports could be attributed to differences in cohort size, patient’s ethnicity, antibody used, and cut-off values. 

At present, the prevalence of PD-L1 expression in Middle Eastern EC has not been explored in detail. Therefore, we conducted this study to determine the expression of PD-L1 in a large cohort of EC and to evaluate the utility of PD-L1 as prognostic biomarker of EC in this ethnicity.

## 2. Materials and Methods

### 2.1. Sample Selection

Archival samples from 440 EC patients diagnosed between 1990 and 2016 at the King Faisal Specialist Hospital and Research Center (Riyadh, Saudi Arabia) were included in the study. Detailed clinico-pathological data were noted from case records and have been summarized in Table 1. Endometrioid endometrial carcinomas were categorized as Type I and all other histologic subtypes were classified as Type II EC. The International Federation of Obstetrics and Gynecology (FIGO) system was used for staging and grading of tumors. Depth of myometrial invasion ≥50% was considered high myometrial invasion, whereas <50% was considered low myometrial invasion. All patients had surgery as their primary treatment. A total of 29.1% (128/440) of patients received radiotherapy alone following surgery. Moreover, 19.1% (84/440) of patients received adjuvant chemotherapy, of which 69 patients received carboplatin and paclitaxel combination whereas 15 patients received carboplatin alone. Overall survival was defined as the length of time from the date of diagnosis that patients diagnosed with the disease are still alive. Recurrence-free survival was defined as the length of time after primary treatment for a cancer ends that the patient survives without any signs or symptoms of that cancer. Disease-free survival was defined as the length of time after primary treatment for a cancer ends that the patient survives without any signs or symptoms of that cancer or death. All samples were obtained from patients with approval from the Institutional Review Board of the hospital. For the study, waiver of consent was obtained for archived paraffin tissue blocks from the Research Advisory Council (RAC) under project RAC# 2180 001 (approved on 7 March 2018).

### 2.2. Tissue Microarray Construction and Immunohistochemistry 

All samples were analyzed in a tissue microarray (TMA) format. TMA construction was performed as described earlier [23]. Briefly, tissue cylinders with a diameter of 0.6 mm were punched from representative tumor regions of each donor tissue block and brought into recipient paraffin block using a modified semiautomatic robotic precision instrument (Beecher Instruments, Woodland, WI, USA). Two cores of EC were arrayed from each case.

Tissue microarray slides were processed and stained manually as described previously [24]. Primary antibody against PD-L1 (E1L3N, 1:50 dilution, pH 9.0, Cell Signaling Technology, Danvers, MA, USA) was used. A membranous and/or cytoplasmic staining was observed. Only the membrane staining was considered for scoring. PD-L1 was scored as described previously [25]. Briefly, the proportion of positively stained cells was calculated as a percentage for each core and the scores were averaged across two tissue cores from the same tumor to yield a single percent staining score representing each cancer patient. For the purpose of statistical analysis, the scores were dichotomized. Cases showing expression level of ≥5% were classified as positive and those with less than 5% as negative. Only staining of tumor cells was considered for percentage calculation.

MMR protein staining and evaluation was done as described previously [26]. Briefly, MMR protein expression was evaluated using MSH2, MSH6, MLH1, and PMS2 proteins. Tumor was classified as deficient MMR if any of the four proteins showed loss of staining in cancer with concurrent positive staining in the nuclei of normal epithelial cells. Otherwise, they were classified as proficient MMR.

IHC scoring was done by two pathologists, blinded to the clinico-pathological characteristics. Discordant scores were reviewed together to achieve agreement.

### 2.3. POLE Mutation Analysis

*POLE* mutation data were available from our previous study [27].

### 2.4. Statistical Analysis

The associations between clinico-pathological variables and protein expression were performed using contingency table analysis and Chi square tests. Survival curves were generated using the Kaplan–Meier method. Multivariate logistic regression analysis was performed to assess the correlation of lymph node metastasis and clinico-pathological variables. Two-sided tests were used for statistical analyses with a limit of significance defined as *p*-value of <0.05. Data analyses was performed using the JMP11.0 (SAS Institute, Inc., Cary, NC, USA) software package.

## 3. Results

### 3.1. Patient Characteristics

Median age of the study population was 59.3 years (range: 26–94 years). The majority of tumors were of Type I (endometrioid adenocarcinoma) histology, accounting for 88.0% (387/440) of ECs, with an almost equal distribution among the three grades. A nearly equal number of cases showed high and low myometrial invasion (50.2% vs. 49.8%). The majority of the cases were Stage I tumors (64.8%; 285/440). MMR deficient tumors accounted for 12.1% (53/440) of ECs in our cohort (Table 1).

### 3.2. PD-L1 Protein Expression in Endometrial Cancer and Its Clinico-Pathological Associations

**Figure 1:**
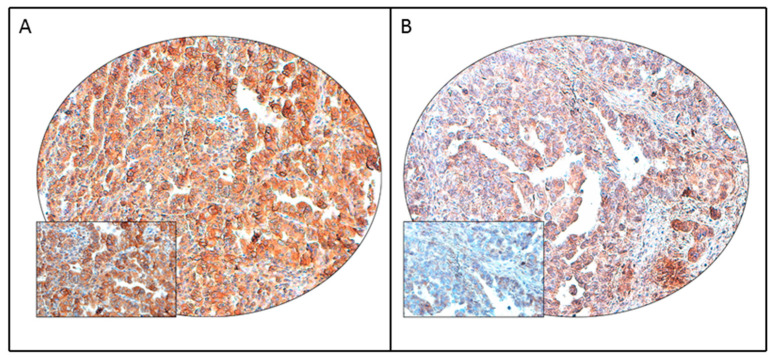
PD-L1 immunohistochemical staining in endometrial cancer tissue microarray (TMA). Representative examples of tumors showing (**A**) high expression and (**B**) low expression (right panel) of PD-L1. (20×/0.70 objective on an Olympus BX 51 microscope. (Olympus America Inc, Center Valley, PA, USA) with the inset showing a 40× 0.85 aperture magnified view of the same TMA spot).

PD-L1 protein expression was analyzed immunohistochemically in 440 EC samples. PD-L1 expression was noted in 18.9% (83/440) of ECs (Figure 1) and found to be significantly associated with Type II (non-endometrioid) histology (*p* = 0.0005) and lymph node metastasis (*p* = 0.0172). However, no association was found between PD-L1 expression and mismatch repair (MMR) immunohistochemistry (*p* = 0.4435) (Table 2). We next analyzed the association between PD-L1 expression and DNA polymerase epsilon (*POLE*) mutation. *POLE* mutation was detected in 0.5% (2/431) of cases in our cohort and was not associated with PD-L1 expression. Both the cases showing *POLE* mutation were negative for PD-L1 (Table 2).

### 3.3. Prognostic Impact of PD-L1 Expression in Endometrial Cancer 

**Figure 2:**
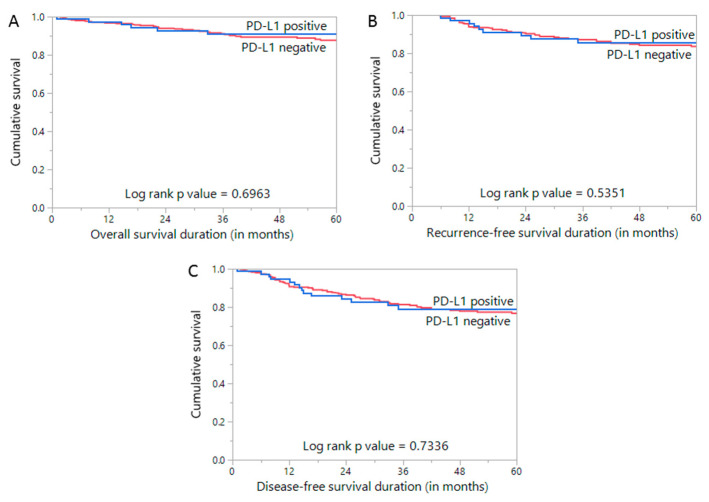
Survival Analysis of PD-L1 protein expression. Kaplan–Meier survival plot showing no statistically significant difference between PD-L1 positive and negative tumors for (**A**) overall survival, (**B**) recurrence-free survival and (**C**) disease-free survival.

We evaluated the effect of PD-L1 expression on overall survival (OS), recurrence-free survival (RFS), and disease-free survival (DFS). However, PD-L1 expression was not associated with OS (*p* = 0.6963), RFS (*p* = 0.5351), or DFS (*p* = 0.7336) (Figure 2A–C). In multivariate logistic regression analysis, PD-L1 expression was an independent risk factor for lymph node metastasis in EC (odds ratio: 2.94; 95% CI: 1.26–6.84; *p* = 0.0123) (Table 3).

## 4. Discussion

Immunotherapies have gained much attention in current oncology practice. In recent years, PD-L1 has shown impressive clinical results in many solid tumors [15,16,28,29,30,31]. Therefore, identification of PD-L1 expression in tumors can offer important value in guiding clinical decisions to use immunotherapy. Different studies have investigated the role of PD-L1 in EC [17,18,19,20,21,22]. However, the results did not reach consensus. Moreover, data about PD-L1 expression in EC from Middle Eastern ethnicity are limited [32,33]. Therefore, in this study, we determined the expression of PD-L1 by IHC in a large cohort of EC and explored its correlation with clinico-pathological features.

In the present study, the incidence of PD-L1 expression was 18.9% in EC patients. This is consistent with previous reports where the identified PD-L1 expression in EC ranges from 14% to 56% [20,32,34,35]. We found a significant association between PD-L1 expression and Type II EC, which is in agreement with previous reports [34,35].

Interestingly, PD-L1 expression was significantly associated with lymph node metastasis and multivariate analysis demonstrated that high PD-L1 expression is an independent marker for lymph node metastasis. In concordance with our results, previously, few studies have reported the correlation between PD-L1 expression and risk of lymph node metastasis [36,37,38]. This finding is of important clinical implication, since PD-L1 expression could be considered as a potential biomarker for lymph node metastasis in EC. Identifying predictive factors for immunotherapeutic agents’ response is of great importance in helping oncologists to select patients who might benefit from immunotherapy. 

MMR deficient status has been found to be a good predictive factor for immunotherapy response [39,40]. Furthermore, previous studies have shown that PD-L1 overexpression is significantly associated with MMR deficient tumors [20,21,41]. Expression of PD-L1 was similar in MMR deficient and proficient tumors in our cohort with no significant differences found in PD-L1 expression in both groups. This finding suggests that PD-L1 has no predictive advantage in MMR deficient EC subgroup and possibly PD-L1 expression status alone should be applied in selecting patients for immunotherapy regardless of their MMR status. A recent study has also shown lack of association between PD-L1 expression and MMR status in a large cohort of Western EC [34]. 

EC patients harboring *POLE* mutation have been shown to offer better immunotherapy response [42]. Therefore, we took advantage of availability of *POLE* mutation data on 431 patients [27] and analyzed the association with PD-L1 expression. Our results confirmed a lack of correlation between PD-L1 expression and *POLE* mutation in this cohort. Our result is contradicting previously reported higher frequency of PD-L1 expression in *POLE* mutant and MMR deficient tumors [43].

There are conflicting results in the literature, not only on PD-L1 expression incidence in EC and their clinico-molecular associations, but also their prognostic value and impact on patient survival. With regards to patient outcome, our results demonstrated that PD-L1 expression is not associated with patients’ overall survival, disease-free survival, or recurrence-free survival. This is in line with a previous study [32] where patients’ survival was not affected by PD-L1 expression in either tumor cells or immune infiltrates. Another large study on 689 EC samples evaluated the expression of PD-L1 on tumor cells and found no association with disease-specific survival, even after stratifying by histologic subtype [34]. Furthermore, a recent large metanalysis with more than 1600 patients suggested that PD-L1 expression is not associated with poor prognosis in endometrial cancer patients. However, they conclude that PD-L1 expression is positively correlated with poor differentiation and advanced stage in endometrial cancer [44]. These findings are in concordance with our study results, where we show that PD-L1 expression is strongly associated with lymph node metastasis. Since lymph node metastasis is an indicator of advanced disease, our study could provide further evidence that PD-L1 might not correlate with overall patient’s prognosis but can be used as a marker for advanced disease in endometrial cancer.

## 5. Conclusions

We detected PD-L1 expression in 18.9% of EC cases. We also demonstrated that PD-L1 was strongly associated with non-endometroid EC. This study also could not reveal any association between PD-L1 expression and patient survival. More importantly, our results show PD-L1 expression to be an independent prognostic marker of lymph node metastasis. Lymph node metastasis is an indicator of advanced disease and our study provides evidence that PD-L1 could be used as a marker of advanced disease in endometrial cancer. More studies are needed to identify patients who might benefit from immunotherapy in EC from this ethnicity, and to determine the role of immunotherapy in EC patients with lymph node metastasis.

## Figures and Tables

**Table 1:** Clinico-pathological variables for the patient cohort (*n* = 440).

Clinico-Pathological Parameter	*n* (%)
Age	
Median	59.3
Range(IQR) ^	53.0–66.2
Histologic subtype	
Type I	387 (88.0)
Type II	53 (12.0)
Myometrial invasion	
High	221 (50.2)
Low	219 (49.8)
Histological grade	
Well differentiated	148 (33.6)
Moderately differentiated	147 (33.4)
Poorly differentiated	130 (29.6)
Unknown	15 (3.4)
pT	
T1	308 (70.0)
T2	55 (12.5)
T3	58 (13.2)
T4	19 (4.3)
pN	
N0	410 (93.2)
N1-2	30 (6.8)
pM	
M0	417 (94.8)
M1	23 (5.2)
Tumor Stage	
I	285 (64.8)
II	48 (10.9)
III	70 (15.9)
IV	37 (8.4)
Primary treatment	
Surgery	440 (100.0)
Adjuvant chemotherapy	84 (19.1)
Paclitaxel and Carboplatin	69 (15.7)
Carboplatin only	15 (3.4)
Adjuvant Radiotherapy	175 (39.8)

^ IQR—inter quartile range; pT—pathologic tumor size; pN—pathologic lymph node metastasis; pM—pathologic distant metastasis.

**Table 2:** Association of PD-L1 protein expression with clinico-pathological characteristics in endometrial cancer.

	Total		PD-L1 Positive		PD-L1 Negative		*p* Value
	No.	%	No.	%	No.	%	
No. of patients	440		83	18.9	357	81.1	
Age (years)							
≤60	236	53.6	42	17.8	194	82.2	0.5387
>60	204	46.4	41	20.1	163	79.9	
Histologic subtype							
Type I	387	87.9	63	16.3	324	83.7	0.0005 *
Type II	53	12.1	20	37.7	33	62.3	
Myometrial invasion							
High	221	50.2	49	22.2	172	77.8	0.0741
Low	219	49.8	34	15.5	185	84.5	
Grade							
Grade 1	148	34.8	22	14.9	126	85.1	0.0893
Grade 2	147	34.6	24	16.3	123	83.7	
Grade 3	130	30.6	32	24.6	98	75.4	
pT							
T1	308	70.0	53	17.2	255	82.8	0.0570
T2	55	12.5	14	25.5	41	74.5	
T3	58	13.2	8	13.8	50	86.2	
T4	19	4.3	8	42.1	11	57.9	
pN							
N0	410	93.2	72	17.6	338	82.4	0.0172 *
N1-N2	30	6.8	11	36.7	19	63.3	
pM							
M0	417	94.8	77	18.5	340	81.5	0.3822
M1	23	5.2	6	26.1	17	73.9	
Tumor Stage							
I	285	64.8	47	16.5	238	83.5	0.2443
II	48	10.9	11	22.9	37	77.1	
III	70	15.9	14	20.0	56	80.0	
IV	37	8.4	11	29.7	26	70.3	
MMR IHC							
dMMR	53	12.1	8	15.1	45	84.9	0.4435
pMMR	387	87.9	75	19.4	312	80.6	
*POLE* mutation							
Present	2	0.5	0	0.0	2	100.0	0.3576
Absent	429	99.5	82	19.1	347	80.9	
5 year overall survival				90.9		87.0	0.6963
5 year recurrence-free survival				85.7		83.6	0.5351
5 year disease-free survival				78.9		76.8	0.7336

* significant *p*-value; IHC—immunohistochemistry; dMMR—deficient mismatch repair; pMMR—proficient mismatch repair; *POLE—DNA polymerase epsilon*.

**Table 3:** Multivariate analysis to assess relationship between lymph node metastasis and clinico-pathological characteristics.

Clinico-Pathological Variables	Odds Ratio	95% CI	*p*-Value
Age			
Age>60 years (vs. ≤60 years)	0.65	0.29–1.47	0.3020
Histologic grade			
Grade 3 (vs. Grade 1–2)	2.14	0.92–4.96	0.0755
Myometrial invasion			
High (vs. Low)	2.84	1.01–7.94	0.0473 *
pT			
T3-4 (vs. T1-2)	2.42	0.90–6.49	0.0801
pM			
M1 (VS. M0)	0.63	0.03–11.74	0.7548
Tumor Stage			
IV (vs I–III)	0.28	0.03–2.75	0.2766
MMR statusd			
MMR (vs. pMMR)	1.20	0.41–3.58	0.7376
PD-L1 expression			
Positive (vs. Negative)	2.94	1.26–6.84	0.0123 *

* significant *p*-value.

## Data Availability

The data presented in this study are available in the article.
